# A multi-omics supervised autoencoder for pan-cancer clinical outcome endpoints prediction

**DOI:** 10.1186/s12911-020-1114-3

**Published:** 2020-07-09

**Authors:** Kaiwen Tan, Weixian Huang, Jinlong Hu, Shoubin Dong

**Affiliations:** Communication & Computer Network Lab of Guangdong, School of Computer Science & Engineering, South China University of Technology, Wushan Road, Guangzhou, 381 China

**Keywords:** Multic-omics, Autoencoder, Fusion, Representation, Pan-Cancer, Endpoints

## Abstract

**Background:**

With the rapid development of sequencing technologies, collecting diverse types of cancer omics data become more cost-effective. Many computational methods attempted to represent and fuse multiple omics into a comprehensive view of cancer. However, different types of omics are *related* and *heterogeneous*. Most of the existing methods do not consider the difference between omics, so the biological knowledge of individual omics may not be fully excavated. And for a given task (e.g. predicting overall survival), these methods prefer to use sample similarity or domain knowledge to learn a more reasonable representation of omics, but it’s not enough.

**Methods:**

For the purpose of learning more useful representation for individual omics and fusing them to improve the prediction ability, we proposed an autoencoder-based method named MOSAE (Multi-omics Supervised Autoencoder). In our method, a specific autoencoder were designed for each omics according to their size of dimension to generate *omics-specific* representations. Then, a supervised autoencoder was constructed based on specific autoencoder by using labels to enforce each specific autoencoder to learn both *omics-specific* and *task-specific* representations. Finally, representations of different omics that generate from supervised autoencoders were fused in a traditional but powerful way, and the fused representation was used for subsequent predictive tasks.

**Results:**

We applied our method over TCGA Pan-Cancer dataset to predict four different clinical outcome endpoints (OS, PFI, DFI, and DSS). Compared with traditional and state-of-the-art methods, MOSAE achieved better predictive performance. We also tested the effects of each improvement, which all have a positive effect on predictive performance.

**Conclusions:**

Predicting clinical outcome endpoints are very important for precision medicine and personalized medicine. And multi-omics fusion is an effective way to solve this problem. MOSAE is a powerful multi-omics fusion method, which can generate both omics-specific and task-specific representation for given endpoint predictive tasks and improve the predictive performance.

## Background

### Introduction

Driven by high-throughput sequencing technologies, many cancer genomics programs have been established to generate omics data, so the cancer data grows almost exponentially in volume, variety and complexity [1]. Among these large-scale sequencing studies, The Cancer Genome Atlas (TCGA) is the most famous one, which generates a rich resource of multi-omics data and provides more than 30 cancer types. In TCGA, each patient holds multi-omics profiling, including DNA methylation, protein expression, gene expression RNASeq, miRNA mature strand expression and so on. These different types of omics are *related* and *heterogeneous*. For a single patient, different omics are associated with the same trait and biological connections exist between different omics, but different omics provide different molecular level information for the same trait and they are also different in quantitative and descriptive forms. Therefore, integration analysis of multi-omics is a great challenge, and powerful integration methods may promote the exploration of pathogenesis of cancer by taking advantage of different omics, and furthermore, accelerating the development of precision medicine and personalized medicine.

In most cancers, the number of patients is only a few hundred, but many omics has tens of thousands of dimensions, which means for a specific task, most omics features are noise which causes the curse of dimensionality problem. Furthermore, if we use multiple high-dimension omics simultaneously, the problem will be more serious. Recently, many works [2] focus on alleviating the curse of dimensionality problem, but in real TCGA datasets, not all omics are high-dimension such as the dimension of protein expression is only a few hundred in the TCGA Pan-Cancer dataset. Moreover, recent works [3] utilize sample similarity to achieve the idea that similar samples should have similar representations, and utilizing domain knowledge to construct associations between and within omics. These methods are powerful in solving clustering problem, but for predicting, the representations that generate from the sample similarity and domain knowledge are not power enough. Because under specific task, there is still a lot of noise (e.g. task-independent information) in these representations, which hinders the performance of these models [4].

Based on above observations, we proposed a model named Multi-omics Supervised Autoencoder (MOSAE), an autoencoder-based multi-omics fusion method, which designs different autoencoder structures for different omics based on dimensional differences and uses label information to enforce autoencoder to equip the ability that the representations generate from autoencoders are associated with subsequent tasks. So each omics will have a specific supervised autoencoder, and the representations produce from the supervised autoencoders will be fused in a simple but powerful manner. Key contributions of this paper are summarized as follows:

1. We observed that different omics contain different biological knowledge, and only a fraction of this knowledge is useful for subsequent tasks. Therefore, we constructed an omics- and task-specific structure of autoencoder (named supervised autoencoder) to explore knowledge of each omics. Each omics has a unique supervised autoencoder, and representations generate from these supervised autoencoder contain both omics- and task-specific biological knowledge.
2. We observed that traditional fusion methods such as concatenation are not suitable for integrating multi-omics, and average is a better alternative. This method averaging the representations by element, hence, knowledge from different omics are enforced to have the same meanings in the same position of dimension.
3. We redesigned the loss function to guarantees the availability of our structure. Specifically, for the supervised autoencoders of each omics, both prediction error and reconstruction error were constructed. And a single prediction error that based on fused representation were constructed. This loss function can prevent information leakage.
4. We verified our method by predicting four different Pan-cancer clinical outcome endpoints. The results shown that MOSAE achieved better results than traditional and state-of-the-art methods, and has robust generalization ability.

### Related work

We summarized recent multi-omics learning methods into two part: representation of omics and fusion between multiple representations of each sample.

For representing of omics, autoencoder is widely used, which is a deep learning method for dimension reduction of high-dimensional omics data [5], representation of cancer patients [6] and even fusion of multi-omics data [7]. And in order to increase the ability of representation of autoencoder, a large number of variants of autoencoder were developed [8]. utilizes nonlinear data self-expressiveness to learn the hidden layer of autoencoder, which is the representation of patient [3]. utilizes feature interaction network and patient similarity network to constrain the training objective of autoencoder that alleviate overfitting and curse of dimensionality problem. Most of above methods are unsupervised or semi-supervised and they perform the same processing on different omics, which reduced the representation ability of autoencoder. Therefore, our method MOSAE employed labels to train autoencoder for each omics and designed the structure of autoencoder according to the characteristics of different omics.

For fusing multiple representations, concatenation is the most common method, which concatenate different omics representations into a single vector directly, but concatenation is often unworkable [1]. Therefore, a plethora of methods have been developed to integrate/fuse multiple omics data. And [9] divides integrative methods into four categories: network-free non-Bayesian (NF-NBY), network-free Bayesian (NF-BY), network-based non-Bayesian (NB-NBY) and network-based Bayesian (NB-BY) methods. They are classified by whether employing a prior on data probability distribution or graphs to model interactions. In NF-NBY, Partial Least Squares (PLS)-based methods are used widely. For example, sPLS [10] is a sparse vision of PLS, and Multi-block PLS [11] performing PLS on a multi-omics dataset. In addition, multi-omics gene-wise weights is another popular method in NF-NBY that integrate different omics into a score for each gene. In NF-BY, iCluster [12] is an innovative method that capture the similarities among different omics by minimize within-cluster variance. In NB-NBY, stSVM [13] utilizes diffusion kernels to random walk on each network with restarts, and Similarity Network Fusion (SNF) [14] fuses patient similarity networks (sample-sample network) iteratively from each omics. Affinity Network Fusion (ANF) [15] is an upgrade of SNF that employ state transition matrix to obtain affinity matrix (similarity matrix) and fused weighted view by a ‘smooth’ procedure. Our method belongs to NB-NBY that no prior is assumed on data and no graph is used for model interactions, which means our model structure is simpler than others. However, our experimental results show that our fusion method outperforms concatenation and some NB-NBY-based methods [3], and we believe our fusion method may contain some real biological meaning.

## Methods

Our method is based on autoencoder so we give a brief introduction to it, and then we divided our method into three part: specific autoencoder, supervised autoencoder and multi-omics fusion, and discussed them in later sections.

### Autoencoder

Autoencoder is an unsupervised neural network method that applies back-propagation, setting the output values to be equal to the inputs. And one of the hidden layer of autoencoder is considered as the representation of the inputs. If the hidden layer has fewer neural units than the input layer, we treat the hidden layer as a compressed knowledge representation of the original input, otherwise we treat the hidden layer as a ‘diversity’ representation that map the original space to a higher dimensional space.

Usually, autoencoder is divided into two processes, encoder and decoder. Suppose the original input is *X* ∈ *ℝ*^*N* × *p*^, a sample-feature matrix with *N* samples and *p* features. An one-layer neural network with parameter *Θ*_*e*_ is regarded as encoder:

$$ Encoder\left(X,{\varTheta}_e\right)=H\in {\mathbb{R}}^{N\times k} $$

*H* is usually referred to as latent representation of input *X*. The encoder maps *N* samples from p-dimension space to k-dimension space. And another one-layer neural network with parameter *Θ*_*d*_ is regarded as decoder:

$$ Decoder\left(H,{\varTheta}_d\right)=\overset{\sim }{X}\in {\mathbb{R}}^{N\times p} $$

$\overset{\sim }{X}$ is reconstruction representation which has the same shape as *X*. The decoder maps *N* samples from k-dimension space back to p-dimension space, and it should be noted that *X* and $\overset{\sim }{X}$ are different. The whole process of autoencoder can be expressed as:

$$ Decoder\left( Encoder\left(X,{\varTheta}_e\right),{\varTheta}_d\right)=\overset{\sim }{X} $$

Finally, the objective function of autoencoder can be formulated with Frobenius norm:

$$ \underset{\varTheta_e,{\varTheta}_d}{\arg\ \min }{\left\Vert X-\overset{\sim }{X}\right\Vert}_F^2 $$

The objective function is also called reconstruction error, which try to penalize the difference between *X* and $\overset{\sim }{X}$. And the latent representation *H* is generally used in subsequent tasks as the representation of input, because it’s widely believed that *H* retains input information in a better form. And there is an interesting fact that if all neural networks in autoencoder is linear and the dimension of *H* is less than the dimension of *X*, we would observe a similar dimensionality reduction as observed in principal component analysis (PCA).

### Specific autoencoder

As mentioned before, different omics have different properties, more specifically, the dimension of some omics are high and other are low. This situation is very common, but current methods are not considered. In order to deal with this situation, we thought that the omics with high-dimension contain more information than the omics with low-dimension, because high-dimension omics contains more genes (or protein), and more genes (or protein) mean more complete descriptions of a patient.

Therefore, for high-dimension omics (hold more information), we need compress them to a lower dimension space. In addition, the compression step can avoid overfitting. On the contrary, for low-dimension omics, the information is less, so we need to map original omics nonlinearity into higher dimension, and produce more nonlinear combinations of original features. Therefore, in our specific autoencoder, we suppose that there are *M* types of omics *X* = {*X*^(1)^, …, *X*^(*i*)^, …, *X*^(*M*)^}, in which ${X}^{(i)}\in {\mathbb{R}}^{N\times {p}^{(i)}}$ represents the *i*^*th*^ omics. And then the *M* types of omics have been divided into high-dimension group *X*^(*high*)^ and low-dimension group *X*^(*low*)^. Without loss of generality they were formulated as:

$$ {X}^{(high)}=\left\{{X}^{(1)},{X}^{(2)},\dots, {X}^{(l)}\right\} $$

$$ {X}^{(low)}=\left\{{X}^{\left(l+1\right)},{X}^{\left(l+2\right)},\dots, {X}^{(M)}\right\} $$

$$ X={X}^{(high)}\cup {X}^{(low)} $$

And autoencoders for each omics were formulated as:

$$ {H}^{(i)}=\left\{\begin{array}{c} Encoder\left({F}_{\uppi^{(i)}}\left({X}^{(i)}\right),{\varTheta}_e^{(i)}\right)\  if\ {X}^{(i)}\in {X}^{(high)}\ \\ {} Encoder\left({G}_{\uppi^{(i)}}\left({X}^{(i)}\right),{\varTheta}_e^{(i)}\right)\  if\ {X}^{(i)}\in {X}^{(low)}\end{array}\right. $$

$$ {\overset{\sim }{X}}^{(i)}= Decoder\left({H}^{(i)},{\varTheta}_d^{(i)}\right) $$

${F}_{\uppi^{(i)}}\left(\bullet \right)$ is a ‘compression’ neural network and ${G}_{\uppi^{(i)}}\left(\bullet \right)$ is an ‘expansion’ neural network, they have formed omics-specific layers. And the decoder structure is same for all omics. *H*^(*i*)^ is the new latent representation of *i*^*th*^ omics that will be used in subsequent tasks, and all *H*^(*i*)^ have the same dimension k. The loss function become:

$$ \underset{\varTheta_e^{(i)},{\varTheta}_d^{(i)},{\uppi}^{(i)}}{\arg\ \min }{\sum}_{i=1}^M{\left\Vert {X}^{(i)}-{\overset{\sim }{X}}^{(i)}\right\Vert}_F^2 $$

In fact, the structures are different in all omics not only in high-dimension or low-dimension omics, because different autoencoder have different parameters.

### Supervised autoencoder

Now, we have got the autoencoders for each omics, but the latent representations produced by those autoencoders may not good enough to represent the omics for a given task. Follow above ideas, high-dimension omics hold more information, but under a given task, only a few information are useful and others are considered as noises. So many methods attempted to enforce autoencoder to learn more specific information but their representation ability is insufficient. And in this paper, we thought that using labels is the best way to do so. Therefore, we reformulated the loss function of the specific autoencoder as:

$$ \underset{\varTheta_e^{(i)},{\varTheta}_d^{(i)},{\uppi}^{(i)},{\varTheta}_p^{(i)}}{\arg\ \min }{\sum}_{i=1}^M{\left\Vert {X}^{(i)}-{\overset{\sim }{X}}^{(i)}\right\Vert}_F^2+\alpha {\sum}_{i=1}^M\mathcal{L}\left(Y,{\overset{\sim }{Y}}^{(i)}\right) $$

$$ {\overset{\sim }{Y}}^{(i)}= Predictor\left({H}^{(i)},{\varTheta}_p^{(i)}\ \right) $$

$Predictor\left({H}^{(i)},{\varTheta}_p^{(i)}\ \right)$ is a supervised neural network with parameter ${\varTheta}_p^{(i)}$ and input *H*^(*i*)^. *Y* is the label vector of given task. And $\mathcal{L}$ is the prediction loss (cross entropy or mean squared error). The loss function encourages that the representation produced by autoencoder should hold omics- and task-specific knowledge, and *α* is used to adjust propensity. From another perspective, the supervised autoencoder is a common supervised deep neural network with reconstruction error, but in our method, we focus on the representation rather than prediction, so we called it supervised autoencoder.

### Multi-omics fusion

After above processing, we can generate a very powerful representation for each omics, and they can be used directly. But different omics describe different aspects about the same trait, the representation of a single-omics is not comprehensive for a trait. Therefore, we used a very simple but powerful way, average, to fuse those representations. In supervised autoencoder, we got *H*^(*i*)^ for *i*^*th*^ omics, and all *M* representations have the same dimension *k*, so we fused them as:

$$ {H}^{(fusion)}=\frac{1}{M}{\sum}_{i=1}^M{H}^{(i)} $$

Again, we used labels to enforce the *H*^(*fusion*)^ contains task-specific information. And the loss function becomes:

$$ \underset{\varTheta_e^{(i)},{\varTheta}_d^{(i)},{\uppi}^{(i)},{\varTheta}_p^{(i)},{\Theta}_f}{\arg\ \min }{\sum}_{i=1}^M{\left\Vert {X}^{(i)}-{\overset{\sim }{X}}^{(i)}\right\Vert}_F^2+\alpha {\sum}_{i=1}^M\mathcal{L}\left(Y,{\overset{\sim }{Y}}^{(i)}\right)+\beta \mathcal{L}\left(Y,{Y}^{(fusion)}\right) $$

$$ {Y}^{(fusion)}= Predictor\left({H}^{(fusion)},{\Theta}_f\ \right) $$

*Y*^(*fusion*)^ is the result produced by a neural network with parameter Θ_*f*_ and input *H*^(*fusion*)^. All labels were processed uniformly in this loss function, so there is no information leakage. And *α*, *β* are used to adjust propensity, they were set to 1 in this paper. Average is a very simple operation of fusion, but we believe it plays an important role: the elements in each *H*^(*i*)^ may have different meanings, but the average operation enforces the elements in the corresponding positions to have the same meanings. The fused representation may represent a higher level of biological information (e.g. pathway) than the molecular level.

**Fig. 1:**
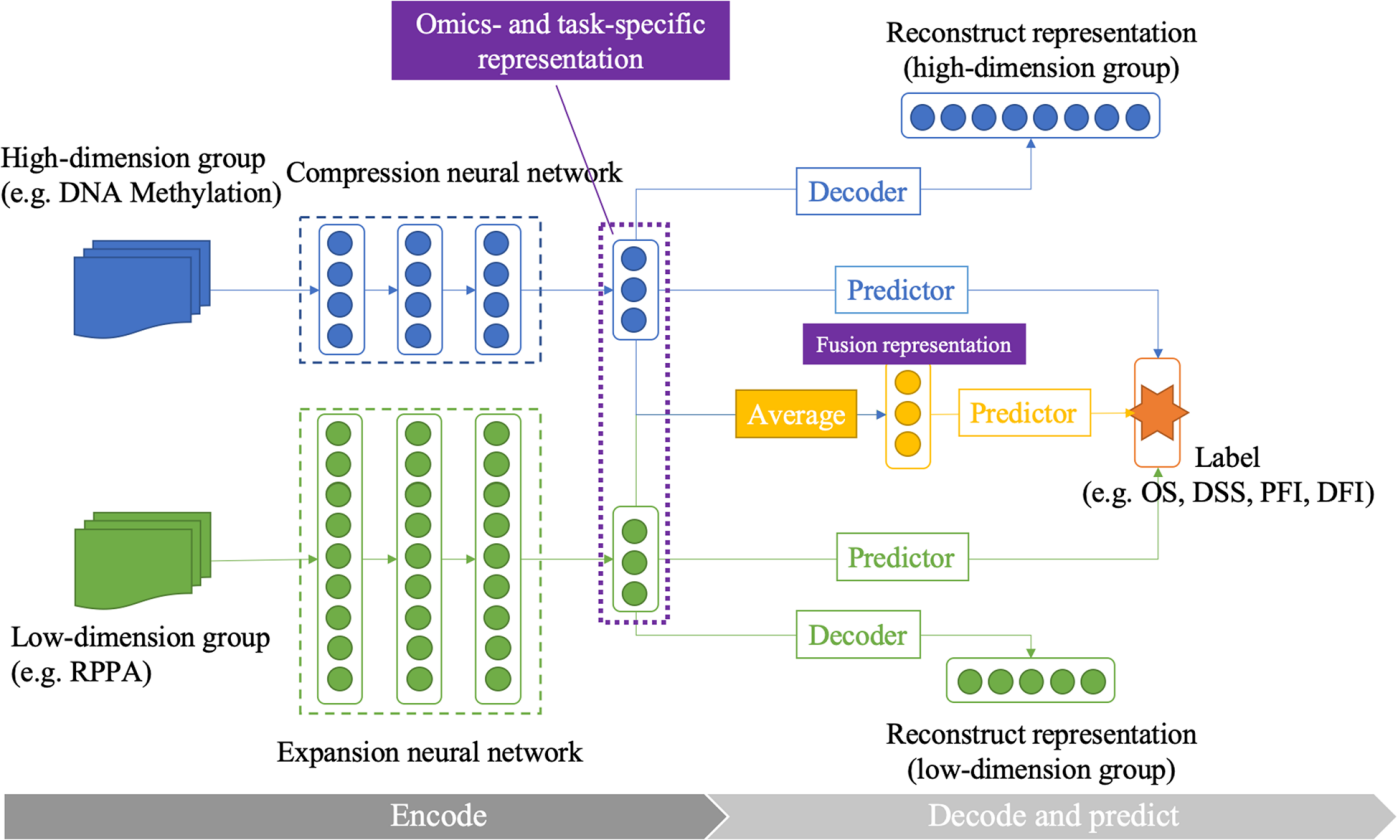
An illustration of MOSAE

An illustration of the whole framework of MOSAE is depicted in Fig. 1. The framework is the same for four different tasks.

## Results

### Dataset

We used TCGA Pan-Cancer data to verify our method, which downloaded from UCSC Xena (https://xenabrowser.net/datapages/). There are many types of omics in TCGA Pan-Cancer, and we selected four of them to verify our method, including DNA methylation, miRNA sequencing (miRNA-Seq), RNA sequencing (RNA-seq) and protein expression (RPPA). Besides, we used four prediction tasks (binary) in our experiment: overall survival (OS), disease-specific survival (DSS), progression-free interval (PFI) and disease-free interval (DFI). They are clinical outcome endpoints, and specific definitions can be found in [16].

For each task, samples with both above four types of omics were selected, and we obtained 5983, 5799, 5983 and 3191 samples for OS, DSS, PFI and DFI, respectively. And for features in each omics types, we removed variables with low variance or low mean and filtered out outliers. Because of the difference between tasks, the number of features in each omics under each task is different. And the final number of features we used is shown in Table 1.

**Table 1:** Details of Preprocessed Data

Omics types	OS	DSS	PFI	DFI
DNA Methylation	4183	4182	4183	4424
miRNA-Seq	658	658	658	680
RNA-Seq	4720	4722	4720	4703
RPPA	210	210	210	210

### Evaluation metric

The proportion of the sample in each task is unbalanced, for example, there are 5983 samples in OS, of which 1692 are positive samples and 4291 are negative samples. Therefore, we selected Area Under the Receiver Operating Characteristic Curve (ROC AUC) as the evaluation metric. In biostatistics, the metric is known as C-index which ranges from 0.5 to 1, and the value over 0.7 indicate that our model may be a good model. We implemented this metric using scikit-learn (https://scikit-learn.org). Besides, we used 5-fold cross validation to calculate the metric, so there is a standard deviation for each result.

### Comparison with other methods

**Fig. 2:**
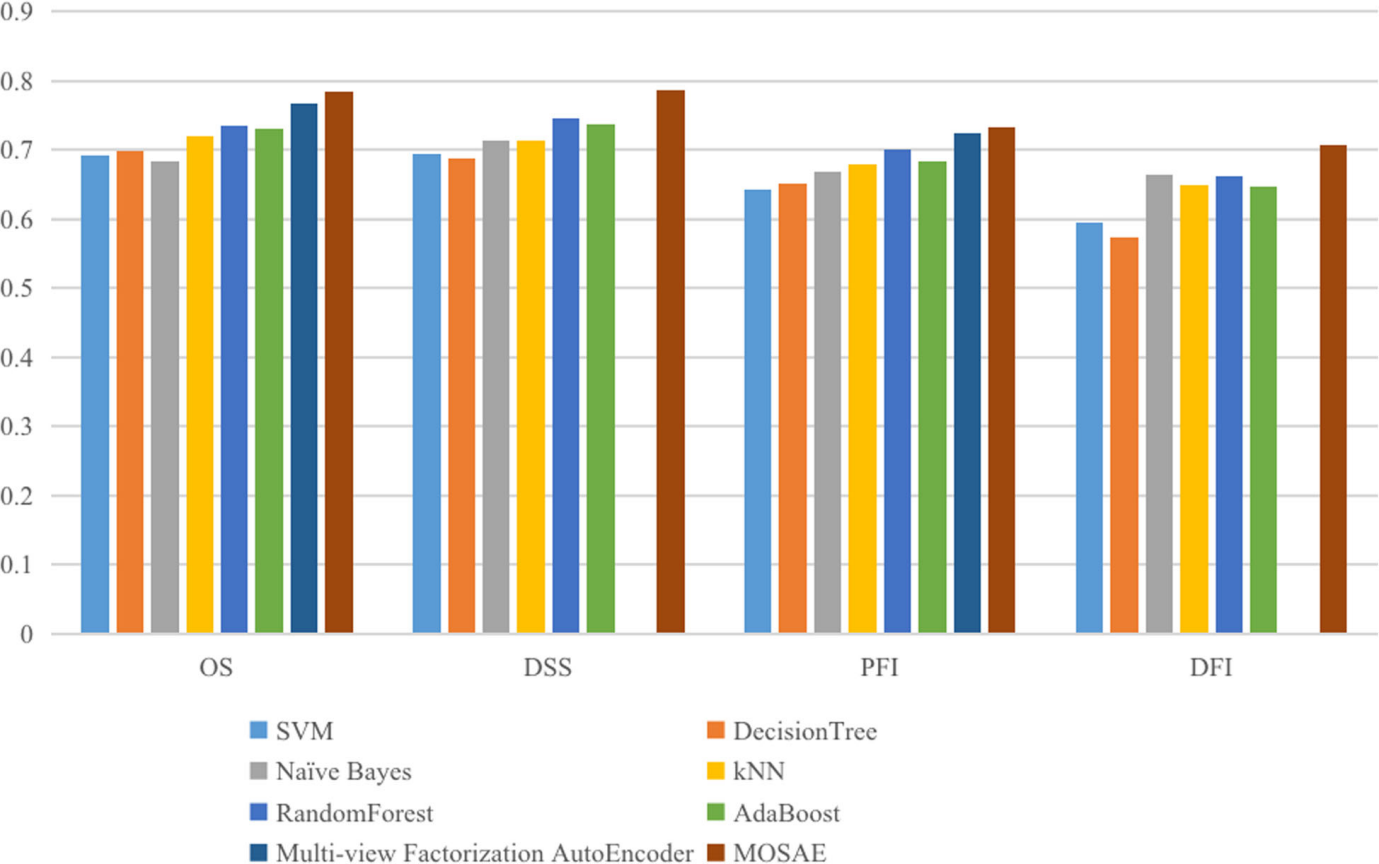
The ROC AUC scores for different tasks and methods

In order to verify the performance of our method MOSAE, we chosen six common machine learning methods as baselines to compare with MOSAE. Those baselines include SVM, DecisionTree, Naïve Bayes, kNN, RandomForest, and AdaBoost. And the input of baselines is the concatenation of multiple omics, and they were implemented using scikit-learn. Multi-view Factorization AutoEncoder predicted OS and PFI using the same dataset and metric as us, and achieved state-of-the-art results. So we used the results in their paper [3] directly to compare with ours. For MOSAE, there are four supervised autoencoders for four omics, and the number of input units is equivalent to the number of input features. DNA methylation and RNA-Seq belong to high-dimension group so the number of the second layer of their corresponding autoencoder was set to 100 as a ‘compression’ neural network, and miRNA-Seq and RPPA belong to low-dimension group so the second layer was set to 1000 as an ‘expansion’ neural network. The latent representation layer was set to 400 for all autoencoders. The ROC AUC scores are shown in Table 2, and the standard deviation is in brackets. At the same time, in order to show the difference between different methods more intuitively, we visualized the ROC AUC scores in Fig. 2. Our algorithm achieves the best results under all tasks.

**Table 2:** Comparison with Other methods

Methods	OS	DSS	PFI	DFI
SVM	0.6905 (±0.0108)	0.6927 (±0.0154)	0.6416 (±0.0119)	0.5950 (±0.0174)
DecisionTree	0.6973 (±0.0082)	0.6877 (±0.0199)	0.6503 (±0.0093)	0.5736 (±0.0276)
Naïve Bayes	0.6825 (±0.0110)	0.7139 (±0.0277)	0.6672 (±0.0074)	0.6631 (±0.0304)
kNN	0.7189 (±0.0086)	0.7134 (±0.0146)	0.6788 (±0.0095)	0.6488 (±0.0474)
RandomForest	0.7355 (±0.0082)	0.7449 (±0.0160)	0.6999 (±0.0134)	0.6621 (±0.0299)
AdaBoost	0.7297 (±0.0042)	0.7369 (±0.0219)	0.6831 (±0.0155)	0.6454 (±0.0254)
Multi-view Factorization AutoEncoder [3]	0.766 (−)	–	0.724 (−)	–
MOSAE	**0.7830** (±0.0081)	**0.7870** (±0.0293)	**0.7325** (±0.0123)	**0.7061** (±0.0393)

### Further performance analysis

In this section, we experimented to examine the performance of various improvements in MOSAE. Since our method is based on autoencoder, we choose a plain autoencoder as the baseline, which is a three layers neural network, and we used the concatenation of multi-omics as input, and used the middle layer as latent representation.

**Fig. 3:**
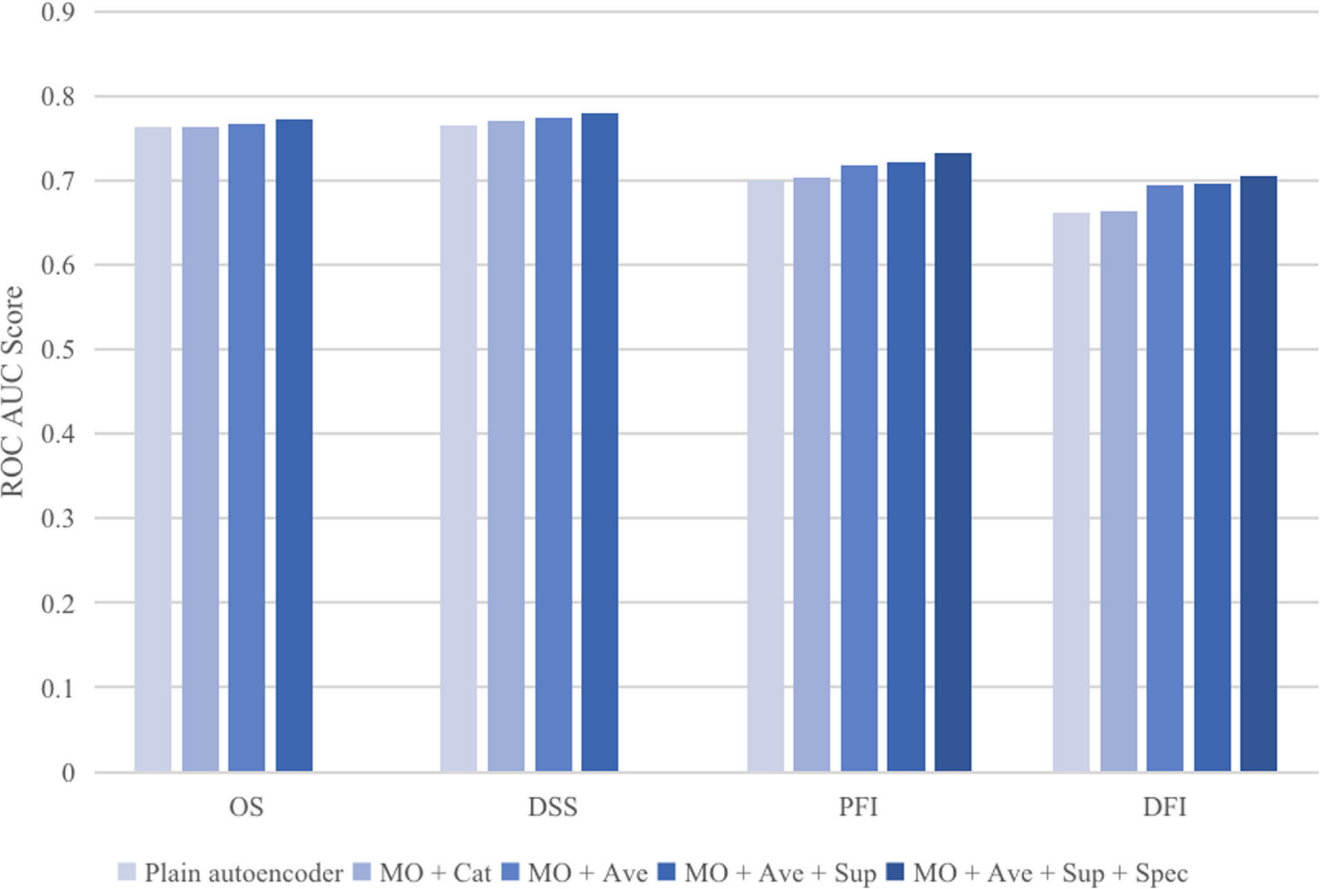
The ROC AUC scores for different Modules

In order to show the results more intuitively, we divide MOSAE into four modules. The first module is multi-omics (**MO**), which mean each omics has its own autoencoder. The second module is fusion, which determines the way latent representations are merged. Here we used two ways, concatenating (**Cat**) and averaging (**Ave**). And for concatenation, the size of each latent representation is set to 100, so the size of fusion representation is 400, which is equivalent to the size of average. The third module is supervised (**Sup**), which means using labels for latent representation. The fourth module is specific (**Spec**), which means the network structure will be adjusted according to the size of the dimension. So MOSAE can be expressed as (**MO** + **Ave** + **Sup** + **Spec**). The ROC AUC scores are shown in Table 3 and visualized in Fig. 3. Every module gives a steady improvement in performance for predicting all tasks.

**Table 3:** Performance of Various Modules of MOSAE

Methods	OS	DSS	PFI	DFI
Plain autoencoder	0.7632 (±0.0058)	0.7660 (±0.0229)	0.6999 (±0.0103)	0.6615 (±0.0340)
MO + Cat	0.7644 (±0.0135)	0.7709 (±0.0292)	0.7030 (±0.0115)	0.6634 (±0.0366)
MO + Ave	0.7682 (±0.0134)	0.7753 (±0.0291)	0.7189 (±0.0136)	0.6942 (±0.0368)
MO + Ave + Sup	0.7721 (±0.0112)	0.7793 (±0.0252)	0.7227 (±0.0124)	0.6960 (±0.0388)
MO + Ave + Sup + Spec	**0.7830** (±0.0081)	**0.7870** (±0.0293)	**0.7325** (±0.0123)	**0.7061** (±0.0393)

## Discussion

### Performance of MOSAE

The results (Table 2, Fig. 2) compared with other methods shown that MOSAE has a significant improvement over the baseline approaches for predicting all tasks, and results are as stable as baselines. Therefore, we can draw a conclusion that our method outperforms traditional methods, and MOSAE can be generalized to different tasks. Besides, we observed that the results of PFI and DFI are worse than OS and DSS. We think this is because PFI and DFI have short-term clinical follow-up intervals. And the amount of data in DFI is much smaller than PFI, so DFI is even worse. In addition, Multi-view Factorization AutoEncoder [3] is an autoencoder-based method, which belongs to NB-NBY methods that utilizes graph to model domain knowledge of features and utilizes sample similarity matrix to fuse different view. But its results for OS and PFI are 0.766 and 0.724, respectively, which is worse than ours (OS: 0.7830 and PFI: 0.7325). This may indicated that our fusion methods and other improvements are more effective than Multi-view Factorization AutoEncoder.

From the results in Table 3 and Fig. 3, we can found that every module gives a steady improvement in performance for predicting all tasks, and **Ave** and **Sup** are the most prominent. The results indicated that the latent representations and fusion representations produced by MOSAE are more powerful for predicting tasks, and average is a better fusion method than concatenation.

### Reconstruction error

From another perspective, MOSAE can be considered as a multi-view neural network with reconstruction error. To examined the role of reconstruction error in MOSAE, we used plain neural network (**NN**) as the comparison object, the input is the same as plain autoencoder (**AE**). And **MO**, **Ave**, **Sup** and **Spec** are the same as above, for example, (**AE** + **MO** + **Ave** + **Sup** + **Spec**) is MOSAE, this is a litter different from above, the purpose is to make a more clearly comparison. And (**NN** + **MO** + **Ave** + **Sup** + **Spec**) means there is an independent neural network for each omics (**MO**), the representations that generate from each neural networks were fused by averaging (**Ave**), those independent neural network were train with labels (**Sup**) and the structure of neural network were dependent on the dimension of omics (**Spec**). From the results (Table 4), we can see that **NN** outperforms **AE**, which means the reconstruction error may not work in predicting, but our **Sup** module changed this situation. This change proves that adding label information to each autoencoder (with reconstruction error) can improve the representation ability. But there is an exception. For OS, reconstruction error did not work well, this may due to the good data quality of OS, which can be confirmed in [16].

**Table 4:** Performance of Reconstruction Error

Methods	OS	DSS	PFI	DFI
NN	0.7641 (±0.0085)	0.7698 (±0.0225)	0.7039 (±0.0134)	0.6631 (±0.0283)
AE	0.7632 (±0.0058)	0.7660 (±0.0229)	0.6999 (±0.0103)	0.6615 (±0.0340)
NN + MO + Ave	0.7726 (±0.0090)	0.7768 (±0.0226)	0.7223 (±0.0157)	0.6880 (±0.0337)
AE + MO + Ave	0.7682 (±0.0134)	0.7753 (±0.0291)	0.7189 (±0.0136)	0.6942 (±0.0368)
NN + MO + Ave + Sup	0.7750 (±0.0083)	0.7747 (±0.0315)	0.7219 (±0.0157)	0.6859 (±0.0268)
AE + MO + Ave + Sup	0.7721 (±0.0112)	0.7793 (±0.0252)	0.7227 (±0.0124)	0.6960 (±0.0388)
NN + MO + Ave + Sup + Spec	**0.7837** (±0.0104)	0.7854 (±0.0301)	0.7299 (±0.0097)	0.7016 (±0.0380)
AE + MO + Ave + Sup + Spec	0.7830 (±0.0081)	**0.7870** (±0.0293)	**0.7325** (±0.0123)	**0.7061** (±0.0393)

## Conclusions

Predicting clinical outcome endpoints are very important for precision medicine and personalized medicine. And multi-omics fusion is an effective way to solve this problem. In this paper, we developed an autoencoder-based method named MOSAE to fuse multi-omics to predict clinical outcome. Firstly, we utilized the difference of omics to design specific structure of autoencoders for different omics, then we employed labels to enforce autoencdoers to learning task-specific representations. Finally, we fused those representations by averaging, which is a simple but powerful operation. MOSAE has been verified in Pan-Cancer dataset, and the results shown that MOSAE outperforms traditional and state-of-the-art methods in all tasks. Every improvement in MOSAE has improved the performance of prediction, and MOSAE has robust generalization ability. Our feature work will focus on designing new structure of autoencoder and developing more powerful and interpretable fusion methods.

## Data Availability

The datasets analysed during the current study are available in UCSC Xena (https://xenabrowser.net/datapages/).

## Abbreviations

MOSAE: Multi-omics Supervised Autoencoder
TCGA: The Cancer Genome Atlas
NF-NBY: Network-Free Non-Bayesian
NF-BY: Network-Free Bayesian
NB-NBY: Network-Based Non-Bayesian
NB-BY: Network-Based Bayesian
PLS: Partial Least Squares
SNF: Similarity Network Fusion
ANF: Affinity Network Fusion
ROC AUC: Area Under the Receiver Operating Characteristic Curve
OS: Overall survival
DSS: Disease-specific survival
PFI: Progression-free interval
DFI: Disease-free interval
